# Dual-modality fibre optic probe for simultaneous ablation and ultrasound imaging

**DOI:** 10.1038/s44172-022-00020-9

**Published:** 2022-07-28

**Authors:** Shaoyan Zhang, Edward Z. Zhang, Paul C. Beard, Adrien E. Desjardins, Richard J. Colchester

**Affiliations:** 1 Department of Medical Physics and Biomedical Engineering, University College London, Gower Street, London WC1E 6BT, UK; 2 Wellcome/EPSRC Centre for Interventional and Surgical Sciences, University College London, Charles Bell House, Foley Street, London W1W 7TY, UK

## Abstract

All-optical ultrasound (OpUS) is an emerging high resolution imaging paradigm utilising optical fibres. This allows both therapeutic and imaging modalities to be integrated into devices with dimensions small enough for minimally invasive surgical applications. Here we report a dual-modality fibre optic probe that synchronously performs laser ablation and real-time all-optical ultrasound imaging for ablation monitoring. The device comprises three optical fibres: one each for transmission and reception of ultrasound, and one for the delivery of laser light for ablation. The total device diameter is < 1 mm. Ablation monitoring was carried out on porcine liver and heart tissue ex vivo with ablation depth tracked using all-optical M-mode ultrasound imaging and lesion boundary identification using a segmentation algorithm. Ablation depths up to 2.1 mm were visualised with a good correspondence between the ultrasound depth measurements and visual inspection of the lesions using stereomicroscopy. This work demonstrates the potential for OpUS probes to guide minimally invasive ablation procedures in real time.

Minimally invasive procedures that perform treatment through small incisions can be invaluable for reaching deep body structures while reducing patient discomfort and recovery times compared to open procedures^1^. One type of treatment commonly performed in a minimally invasive manner in several clinical contexts is tissue ablation. With these treatments, the target tissue is heated or frozen using a variety of techniques to destroy abnormal tissue. This ablation can be carried out in a variety of ways^2^, including electromagnetic thermal techniques, which have been widely used for minimally invasive surgeries^3,4^, and laser ablation, which has proven to be effective in clinical scenarios such as atrial fibrillation ablation^5^ and several types of tumour resection^4,6,7^. Atrial fibrillation (AF) is an application of particular interest as it affects > 12% of the population aged 65 and above^8^. For minimally invasive treatment of AF with ablation, invasive imaging probes that are used for monitoring must have small lateral dimensions and a high degree of mechanical flexibility for integration into catheters. Previously, optical fibres have been used to deliver laser energy for ablation, providing high flexibility to reach high risk and deep-seated targets within the body^9^ and high compatibility with different image guidance modalities during surgery^10^.

Despite its frequent use, challenges still exist to the clinical application of laser ablation, such as the maintenance of tissue coagulation and potential risk of damage on surrounding healthy tissue due to overheating^11^. Thus, real-time monitoring strategies play a significant role for laser treatment. One method to monitor the heating is MRI, which can map the temperature increase with high resolution, allowing optimisation of the ablation plan during the procedure^4,12,13^. However, the use of MRI is limited by the machine availability and high cost^10^. In addition, the low frame rate of MRI imaging limits the real-time monitoring of tissue heating^14^. An alternative is ultrasound thermometry and lesion imaging, which have been widely applied due to their low cost, time saving and simplicity^10^. With ultrasound thermometry, the temperature dependent parameters are employed to reflect the temperature change. For instance, the heating can result in protein denaturation^15^ and coagulative necrosis^16^, which result in changes to the sound speed, thermal expansion and the back-scattered echo signal^10^. However, current ultrasound thermometry techniques only work precisely within a narrow temperature range (43–45 °C)^17^. For ultrasound lesion imaging, contrast is based on the tissue echogenecity change due to bubble formation^14^ and tissue stiffness change^18^ during heating. How-ever, it is challenging to visualise ablated lesions with conven-tional non-invasive ultrasound imaging, especially in the absence of bubble formation for enhancing image contrast^14^.

As an alternative to external ultrasound imaging, interventional ultrasound probes such as endoscopic ultrasound (EUS) and intravascular ultrasound (IVUS) have been successfully used to guide laser ablation during minimally invasive procedures for tumour ablation^6^ and cardiac ablation^19^. However, using electrical ultrasound transducers in interventional scenarios remains a challenge. For instance, due to the difficulty of aligning the imaging plane with the ablation target, current interventional ultrasound probes can only visualise the highly echogenic area around the laser fibre rather than the precise extension of ablation lesion^6^. Additionally, the fabrication of piezoelectric components and electrical circuits on a small scale can result in high complexity and cost^20^. This is especially problematic when broad bandwidth ultrasound waves are required to provide high-resolution imaging. Likewise, it is challenging to fabricate miniaturised ultrasound receivers with broadband reception sensitivity using piezoelectric materials^20^. Moreover, electrically based ultrasound probes are susceptible to electromagnetic interference and not compatible with other modalities such as MRI^21^. Optical techniques, including optical coherence tomography (OCT)^22–24^ have been suggested for ablation monitoring, however, these studies are currently limited to the laboratory and have limitations including the achievable imaging depth.

An emerging imaging technology well-suited for minimally invasive imaging is all-optical ultrasound (OpUS). Unlike conventional ultrasound, which is generated and received electrically via the piezoelectric effect^25^, all-optical ultrasound uses light to both transmit and receive ultrasound^21,26–28^. OpUS can provide broad transmission bandwidth and high reception sensitivity for high-resolution interventional imaging^26^. In addition, the use of optical fibres to generate and receive ultrasound allows for a high level of device miniaturisation with dimensions that are suitable for integration into catheters and needles.

With OpUS, ultrasound waves are generated optically via the photoacoustic effect when the modulated or pulsed light is absorbed within a photoacoustic material, in which the subsequent heating results in a corresponding pressure rise which propagates as an ultrasound wave^29^. Several ultrasound generating materials have been proposed. Polydimethylsiloxane (PDMS) composites are commonly used due to the high thermal expansion coefficient of PDMS, which provides high ultrasound pressures^26^. However, PDMS is transparent at commonly used laser wavelengths, and as such, requires the integration of an optically absorbing material. Various materials have been used for this purpose, including carbon nanotubes^30–33^, carbon black^34^, candle soot^35–37^, organic dyes^38^, quantum dots^39^, metallic nanoparticles^40–42^ and graphene^43^.

Ultrasound reception is performed optically using interferometric acoustic sensors, which can detect the presence of ultrasound at the optical fibre tip^44,45^. Among these sensors, plano-concave micro-resonators that detect acoustically induced deformations of a polymer optical cavity are highly suited for the OpUS imaging, offering high sensitivity, broad reception bandwidth (~20 MHz) and small element size (125 μm o.d)^46^. Significantly, OpUS has successfully achieved two- and three-dimensional imaging alongside in vivo M-mode imaging, proving its feasibility for minimally invasive medical imaging^20,47,48^. Further, while OpUS has not previously been used for laser ablation monitoring, it has been demonstrated for radiofrequency ablation monitoring in a benchtop setup^49^. However, this device required linear scanning of the ultrasound transmitting element and was not readily translatable to a miniature device for clinical application.

An alternative to this type of scanned two-dimensional imaging is to use M-mode imaging. With M-mode ultrasound imaging, consecutive A-lines are concatenated such that the displayed image represents changes in the reflected signal over time^47^. This allows for the use of miniaturised single-element devices and is well-suited to applications where dynamic movements ahead of the probe must be tracked. By combining the optical fibres used for optical ultrasound generation and reception with an optical fibre for laser light delivery for ablation, a highly miniaturised device for synchronous M-mode imaging and ablation can be conceived. Moreover, since the device is all-optical it will be immune to electromagnetic interference and compatible with MRI. Further, it offers the potential for low cost materials and manufacturing processes that are well-suited for single use devices.

In this work, we developed a dual-modality device combining optical ablation and M-mode OpUS imaging to enable real-time monitoring of lesion formation in during laser ablation. The device comprises three optical fibres; one for transmission of ultrasound, one for reception of ultrasound and one for the delivery of laser light for ablation. The total device diameter was < 1 mm, making it compatible with a wide range of minimally invasive devices. Ex vivo porcine heart and liver tissue were used to demonstrate the device performance and a range of laser ablation parameters were tested for both contact and non-contact ablation regimes. The non-contact regime was used to assess the capability of OpUS for accurately tracking the ablation depth with various energy deposition conditions. The contact regime was designed to demonstrate that OpUS could be applied to both coagulated and carbonised lesions. Further, the ablation depth was tracked automatically using a programme based on image segmentation. The depth of the lesion measured by OpUS imaging was compared to microscope images taken post-ablation.

## Results

### Non-contact regime

**Fig. 1:**
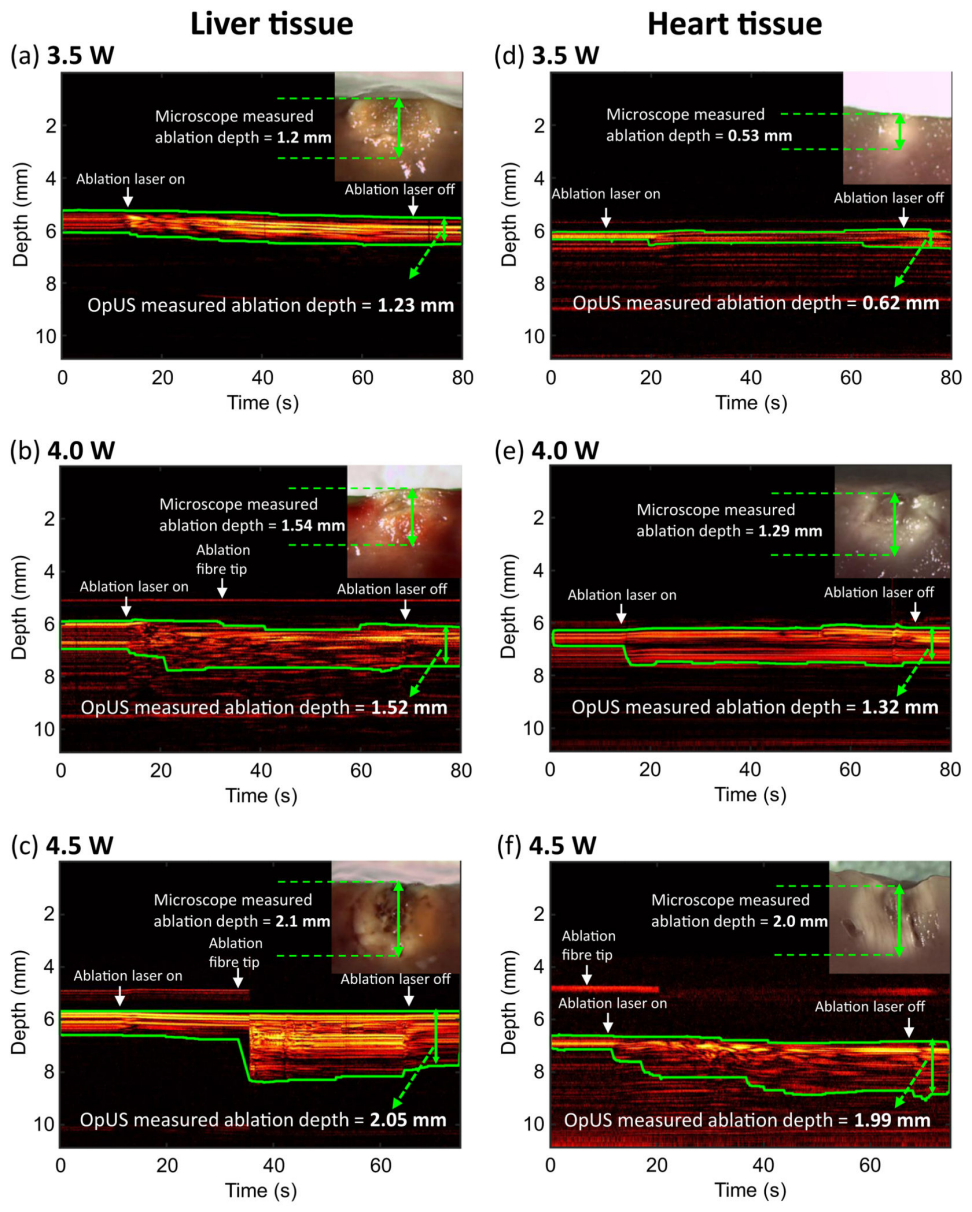
Non-contact regime M-mode OpUS images of lesion formation. The depth axis of the M-mode image has been cropped for display purposes as ablation depths did not exceed those shown. The vertical length of the green boundaries indicates the ablation depth at different times. The laser duration was 60 s. Inset: Corresponding microscope images of the cross-section of the resulting ablated lesion. **a** Liver tissue, laser power: 3.5 W. **b** Liver tissue, laser power: 4.0 W. **c** Liver tissue, laser power: 4.5 W. **d** Heart tissue, laser power: 3.5 W. **e** Heart tissue, laser power: 4.0 W. **f** Heart tissue, laser power: 4.5 W.

M-mode imaging was carried out for both ex vivo porcine heart and liver tissue (Fig. 1). M-mode OpUS imaging provided visualisation of the tissue surface throughout the procedure in all cases. Prior to ablation, the tissue surface was visible as a bright boundary on the M-mode image. When the ablation laser was switched on, the tissue contrast changed immediately as the tissue was heated. Particularly, distinct contrast changes were visible in the M-mode image (Fig. 1c) around 35 s.

Throughout the ablation period the lesion grew in depth which was visible on the M-mode image as increasing brightness and changing contrast in the subsurface region of the tissue. After switching off the laser, the highly echogenic region and image contrast stabilised, from which the completing ablation depth was measured. The lesion boundary with highly echogenic pixels was tracked using the segmentation algorithm, which was displayed as a green boundary on the M-mode images (Fig. 1).

**Fig. 2:**
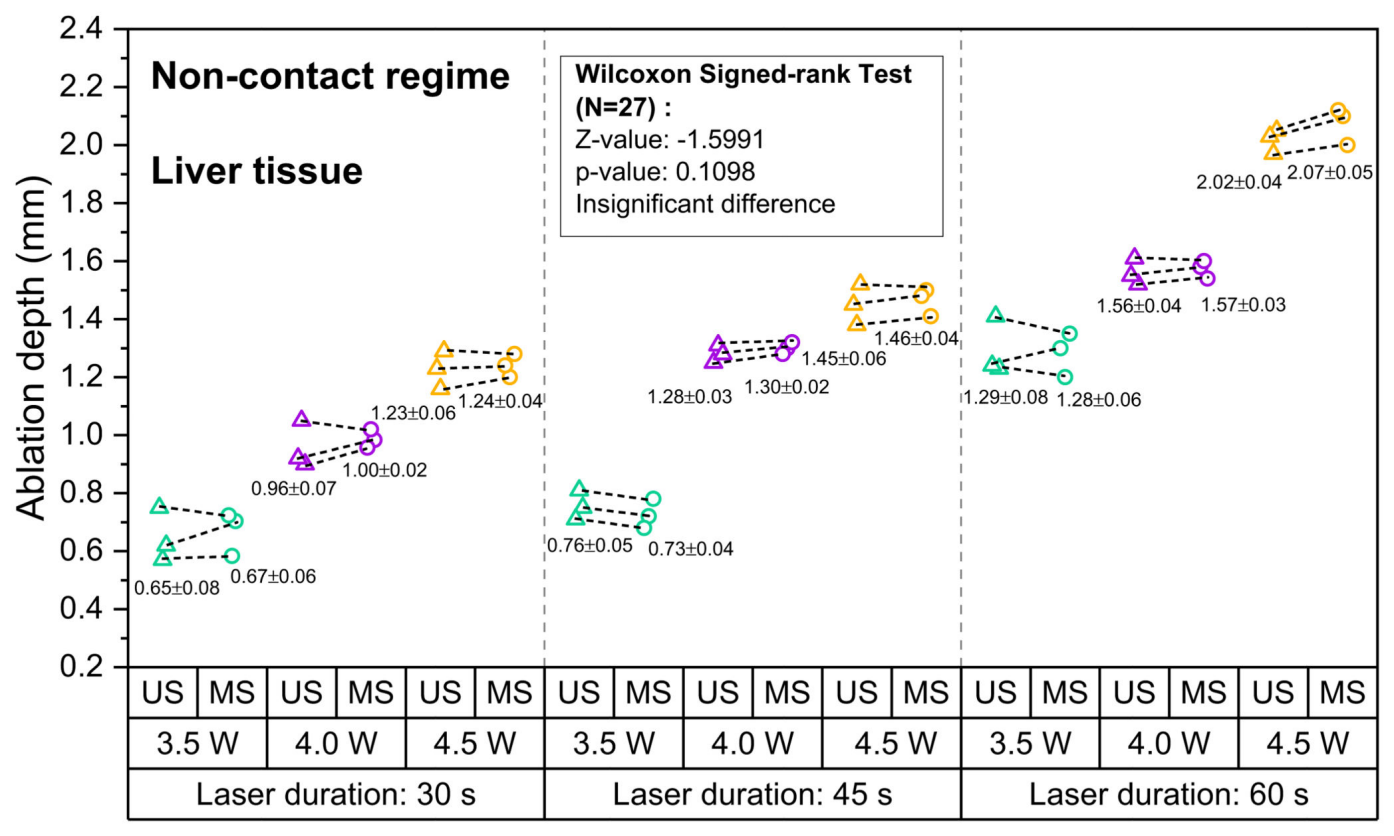
Measured ablation lesion depth in ex vivo porcine liver tissue for the non-contact regime. The resulting ablation depth measured by OpUS imaging (US, triangle) and stereomicroscopy (MS, circle), with different laser duration and power (Green: 3.5 W, Purple: 4.0 W, Yellow: 4.5 W), and Wilcoxon signed-rank test results between the OpUS (US) and stereomicroscopy (MS) measurements, for non-contact laser ablation on liver tissue.

**Fig. 3:**
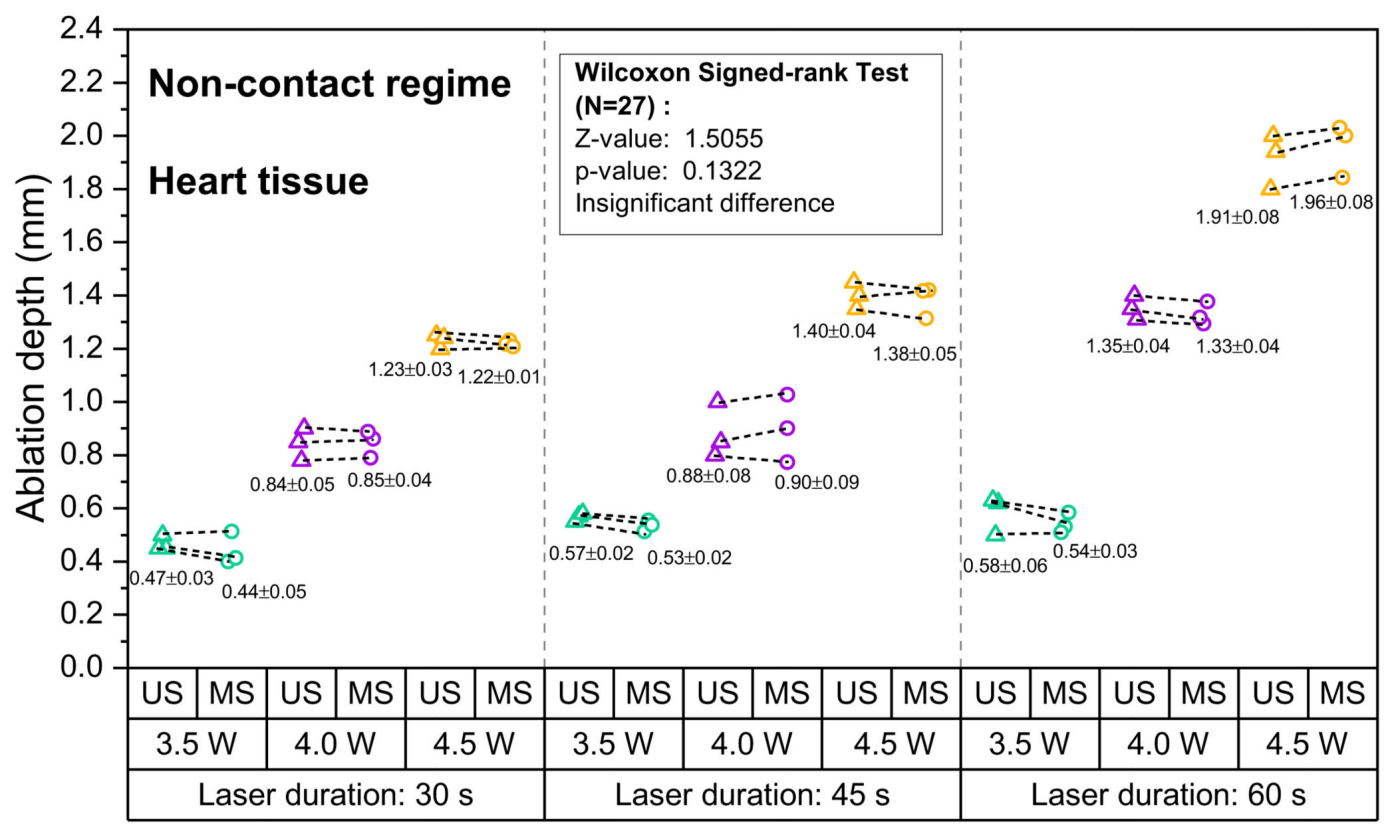
Measured ablation lesion depth in ex vivo porcine heart tissue for the non-contact regime. The resulting ablation depth measured by OpUS imaging (US, triangle) and stereomicroscopy (MS, circle), with different laser duration and power (Green: 3.5 W, Purple: 4.0 W, Yellow: 4.5 W), and Wilcoxon signed-rank test results between the OpUS (US) and stereomicroscopy (MS) measurements, for non-contact laser ablation on heart tissue.

Lesion depth as measured with OpUS increased for longer exposure times and higher laser power for both liver (Fig. 2) and heart tissue (Fig. 3). Lesion size ranged from 0.58 mm at a laser power of 3.5 W and exposure time of 30 s to 2.10 mm for a laser power of 4.5 W and an exposure time of 60 s for liver tissue. For heart tissue, both the minimum (0.41 mm) and maximum (2.03 mm) lesion size were smaller than for liver tissue. For both tissue types, the lesion depths were consistent for repeats of the same exposure time and power. For all laser exposure conditions, the standard deviations of ablation depths measured by OpUS and stereomicroscopy were less than 0.1 mm.

All depth measurements acquired using OpUS imaging were compared to lesion images acquired using stereomicroscopy (Fig. 1 inset). The measurements showed good agreement for both liver (Fig. 2) and heart tissue (Fig. 3). To confirm the agreement, a Wilcoxon Signed-Rank Test was carried out across the acquired data. The test indicated that the difference between ablation depth measured by OpUS and stereomicroscopy was statistically insignificant (Liver tissue: *Z* = − 1.5991, *p* = 0.1098; Heart tissue: *Z* = 1.5055, *p* = 0.1322), demonstrating the efficacy and accuracy of the M-mode OpUS depth measurements.

### Contact regime

**Fig. 4:**
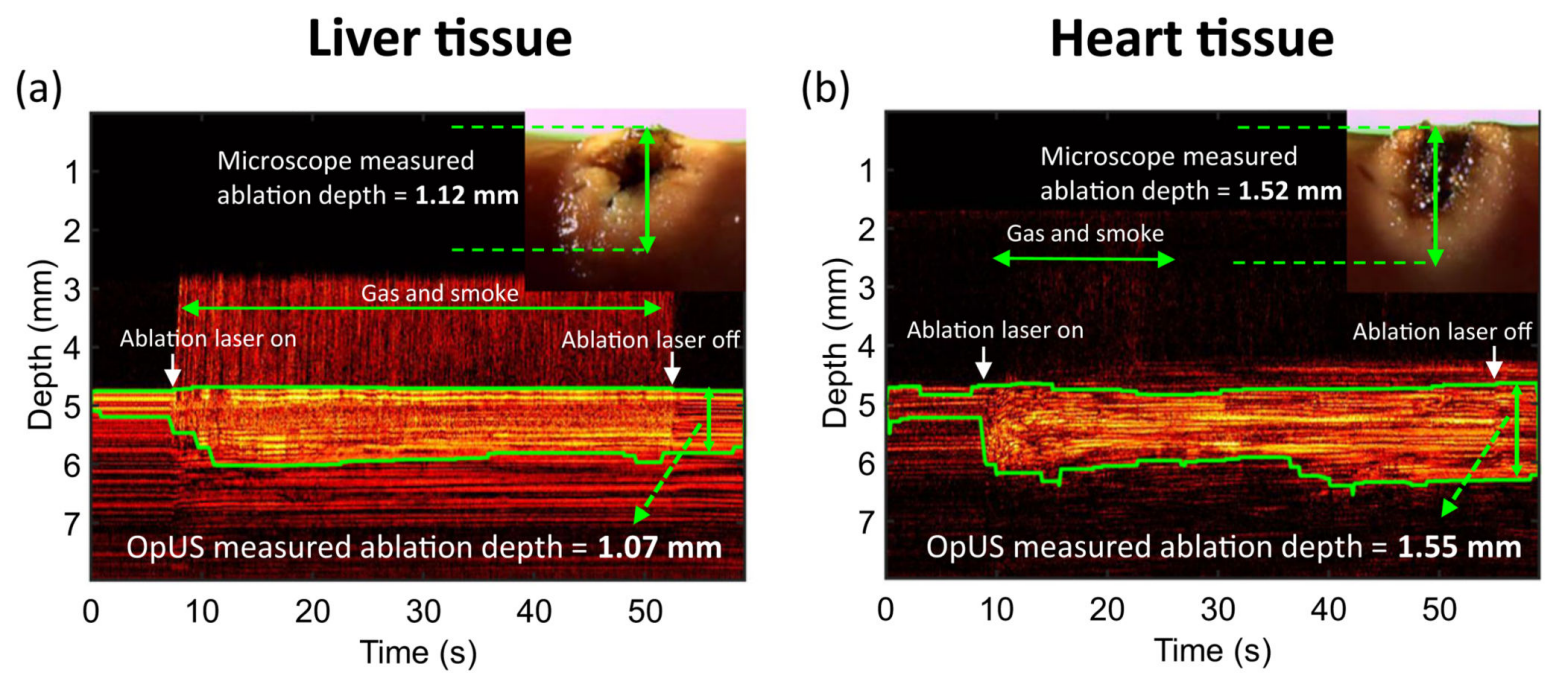
Contact regime M-mode OpUS images of lesion formation. The depth axis of the M-mode image has been cropped for display purposes as ablation depths did not exceed those shown. The applied laser power and duration were 1.0 W and 45 s. Inset: Corresponding microscope images of the cross-section of the resulting ablated lesion. **a** Liver tissue. **b** Heart tissue.

Similar to the non-contact regime, M-mode OpUS imaging provided visualisation of the tissue surface and lesion formation throughout the ablation procedure in all cases. The tissue surface was observed on the M-mode images as a bright boundary prior to ablation (Fig. 4). When the ablation laser was turned on, the brightness of the subsurface region of the tissue changed. Further, a bright signal appeared above the tissue surface (Fig. 4a). This was believed to be caused by ultrasound reflections from gas, bubbles and smoke ejected from the tissue surface due to tissue vaporisation and carbonisation during ablation.

Despite the material ejection from the tissue, good lesion visualisation on the M-mode images was maintained throughout the ablation. After turning off the laser, the image contrast stabilised and the brightness of tissue was significantly distinct from that prior to the ablation. The green boundary that indicated the ablation depth was delineated by segmenting the high brightness regions on the M-mode images. As seen in the microscopy image of lesions (Fig. 4 inset), the area closest to the ablation fibre tip was carbonised. Additionally, the tissue was coagulated and visualised as whitening of colour in the area beyond the carbonisation region. The ablation depth measured by OpUS (Liver: 1.07 mm; Heart: 1.55 mm) and stereomicroscopy (Liver: 1.12 mm; Heart: 1.52 mm) showed good consistency.

## Discussion

In this work, we present a miniaturised dual-modality all-optical probe to carry out optical ablation therapy and lesion imaging concurrently. The device comprised three optical fibres to provide ultrasound transmission, ultrasound reception and excitation light for laser ablation. The ultrasound transmitter had an aperture of 400 *μ*m. The aperture size used provided a narrow ultra-sound beam, giving good lateral resolution for M-mode imaging^43^, which is well-suited for in vivo imaging where there is limited space for device placement. The plano-concave micro-resonator used provided high sensitivity allowing for good signal-to-noise in the acquired images and penetration depth. Laser excitation light for ablation was delivered via a 400 *μ*m core optical fibre with a numerical aperture of 0.22, providing a low optical beam divergence. The advantage of the low divergence is two-fold; high power is maintained due to the reduced beam spreading, and the laser ablation can be confined to a small region, reducing the risk of damaging healthy tissue. The use of optical fibres to provide light for ultrasound imaging and laser ablation allowed for a high degree of miniaturisation (OD < 1 mm) and mechanical flexibility. These are key criteria when considering applications to minimally invasive surgery. Crucially, the device provided stable imaging throughout all procedures, even in the presence of heating caused by the ablation process. In addition, the ablation fibre tip was visible around 1 mm above the tissue surface (Fig. 1b, c, f), which can enable guidance for device placement during the treatment. However, the signals from fibre tip were not always visible due to the weak reflections making it intermittent, depending on probe location and manipulation during the procedures and dynamic range of images.

In order to demonstrate ablation monitoring, liver and heart tissue were chosen due to their clinical relevance^4–7^, as well as their well characterised optical and thermal properties^50,51^. It was found that for the same laser power, liver tissue was more easily ablated than heart tissue, forming larger lesions in the same ablation period (Figs. 2 and 3), especially at lower laser powers. This might result from the differences in optical and thermal properties between liver and heart tissue^50–53^. For instance, a higher optical absorption would lead to more energy deposited on tissue and the thermal diffusion through the tissue will affect the overall lesion size and maximum temperature reached^11^. In some cases the tracked lesion depth decreased after the ablation laser is switched off (e.g., Fig. 1b, c). This is likely due to relaxation of the tissue due to cooling.

For the non-contact ablation regime lesion size increased with increasing laser power and ablation duration (Figs. 2 and 3), as expected from the increased energy deposition. This demon-strates the importance of knowing which ablation parameters to use to achieve the desired result. However, as found here, while ablation lesion sizes were consistent for the same tissue section, the lesion depth varied between the heart tissue and liver. This uncertainty in power deposited compared to lesion size is likely to be worse in vivo, where variations in tissue property and conditions can be large. Tracking of the lesion depth in real-time mitigates this problem. The device presented here was well-suited for this, tracking the full depth of the formed lesion in all cases.

During optical fibre delivered ablation procedures, tissue damage occurs in two stages^54,55^. Initially, the light energy absorbed by the haemoglobin within the tissue leads to a temperature rise and tissue coagulation, which was indicated in the non-contact regime results where no tissue carbonisation was observed (Fig. 1a, b, d, e, f inset). Following the coagulation, when the energy deposition was high enough, carbonisation of coagulated tissue occurred, leading to a sudden contrast change as seen at *ca*. 35 s in the OpUS M-mode image (Fig. 1c). This was confirmed after the experiments with stereomicroscope images (Fig. 1c inset). However, for in vivo tissue ablation in the presence of blood, a coagulum will form around the fibre tip when the light energy is absorbed by the surrounding blood. This is more akin to the contact regime used here, where a carbonised layer formed on the optical fibre tip. In a previous study, the temperature of the fibre tip when coagulum formed was found to be in the range 70–80 °C^56^. Following this stage, the coagulum around the fibre tip will be carbonised, forming a layer of carbon black. This results in a high optical absorption and the majority of light energy will be absorbed within this layer, inducing large temperature increases ( > 300 °C) at the fibre tip^56^. For this case, the dominant mechanism of photothermolysis damage in tissue is by direct contact between tissue and the high-temperature fibre tip, resulting in tissue carbonisation, as seen here in the contact regime (Fig. 4 inset). The lesion size and ablation outcomes in the contact regime were less predictable and consistent even with the same light energy deposition due to the extremely high temperature at the fibre tip. Further, in these experiments the resulting gas, bubbles and smoke formation above tissue surface due to tissue photothermolysis and carbonisation gave rise to image contrast and was visualised as ultrasound backscatter on the M-mode OpUS images (Fig. 4). The ability to distinguish the onset of extreme temperature rises and carbonisation is valuable for clinical imaging, where it can be used to prevent unwanted tissue damage. Further, when the tissue surface is carbonised, the high light absorption and high impedance of the carbonised tissue surface make it difficult for the underlying tissue layer to be ablated^57^. To improve the detection of this effect, future segmentation algorithms can be developed to identify the onset of tissue carbonisation. Additionally, when designing a preclinical catheter, the device can be recessed within some tubing, such that when the tubing contacts the tissue wall some distance is maintained between the ablation fibre and the tissue. Then saline flushing through the tubing can be used to prevent the build up of coagulum, as well as to provide cooling to the device during ablation.

The consistency between the ablation depth measured by OpUS imaging and microscopy (Figs. 2 and 3 inset) found in this work demonstrates the efficacy and feasibility of this imaging paradigm for providing accurate real-time treatment feedback and evaluation of ablation outcomes. The maximum lesion depth visualised was 2.1 mm, which is promising for tracking full lesion depth. This depth was limited by the size of the lesions formed. It is expected that greater depth visualisation will be possible due to the high sensitivity of the OpUS transducer that is comparable to those previously presented, which have demonstrated imaging depths > 2 cm^47^. The segmentation method proposed in this work was robust for delineating lesion boundary on M-mode images (Figs. 1 and 4). While good agreement between the microscope measurements and those found with OpUS was demonstrated, it should be noted that the small sample size will impose a strong limitation on the statistical power.

Several modifications to the probe could allow for therapy, imaging, and sensing with multiple modalities. For this study, the optical fibres did not have a housing. However, for future work the device can be integrated into a medical needle or catheter for preclinical application. The use of silica-based optical fibres and optical technologies means the device was immune to electromagnetic interference, which would allow for use with other imaging modalities such as MRI to improve surgical guidance. Further, the OpUS generating coating can be engineered with composite materials that have wavelength-selective absorption which could allow for imaging and therapy to be implemented through a single multimode fibre, thereby integrating therapy and ultrasound generation while decreasing the lateral device dimensions and increasing the mechanical flexibility. As previously reported, several materials can realise high optical absorption at a specific wavelength (532 nm^39^ and 1064 nm^58^) for ultrasound generation, and low optical absorption at another wavelength (e.g., 808 nm) for transmission of laser light for optical ablation. In addition, attributed to the high degree of device miniaturisation, multiple sensing and imaging techniques can be coupled with the probe within the medical catheter, offering comprehensive guidance for optical ablation treatment. For instance, a fibre-optic temperature sensor^59^ could be integrated into the probe to provide temperature feedback, maintaining the temperature in a range that only allows the tissue to be coagulated rather than carbonised. Alternatively, complementary imaging modalities could be incorporated to provide further lesion characterisation, such as optical coherence tomography (OCT)^23^, which has previously been used to track the development of ablation lesions over much shorter timescales, or photoacoustic imaging (PAI)^60^, which has been used to monitor the temperature change of lesions during photothermal therapy.

This proof-of-concept study demonstrates the potential for OpUS to track laser ablation of tissues. This study focused on the application of laser light to the tissue surface, using heart and liver tissue, which are relevant for applications in atrial fibrillation and cancer resection. However, this work could be extended in future studies to include other modes of ablation, such as laser interstitial thermal therapy (LITT) where the laser applicator is inserted into the tumour to deliver energy for treatment. For effective LITT treatment of malignant tumours, accurate tracking of the resulting thermal damage is required^61^. An advantage of M-mode OpUS in this context is the potential to visualise structures at depths > 1 cm beneath the tissue surface. Further, while the device here was configured to view ahead of the optical fibres, it could be modified to view sideways^62^, thus enabling imaging of vessel walls in intravascular scenarios. This may be relevant for coronary percutaneous intervention, in which intravascular ultrasound (IVUS) can be used to guide the percutaneous laser atherectomy for removing plaque from blood vessels^63^.

The optical fibre-based device introduced in this work integrated therapy and imaging into a small form factor, providing laser excitation for tissue ablation while using optical ultrasound imaging to track ablation depth. The ex vivo experiments on heart and liver tissue demonstrated the feasibility of using M-mode OpUS imaging to track ablation depth in real-time. Crucially, to the authors’ knowledge, this is the first time a miniaturised fibre optic device has been used to provide M-mode OpUS imaging in real-time for ablation monitoring. It is expected that a preclinical device will be developed to show the efficacy of this system in an in vivo context. The dual-modality OpUS device has the potential to guide various minimally invasive procedures such as tumour ablation and atrial fibrillation ablation, by providing ablation depth monitoring in real-time from within a medical needle or catheter. This could improve the accuracy and outcomes of future medical interventions.

## Methods

### Dual-modality ablation and imaging system

**Fig. 5:**
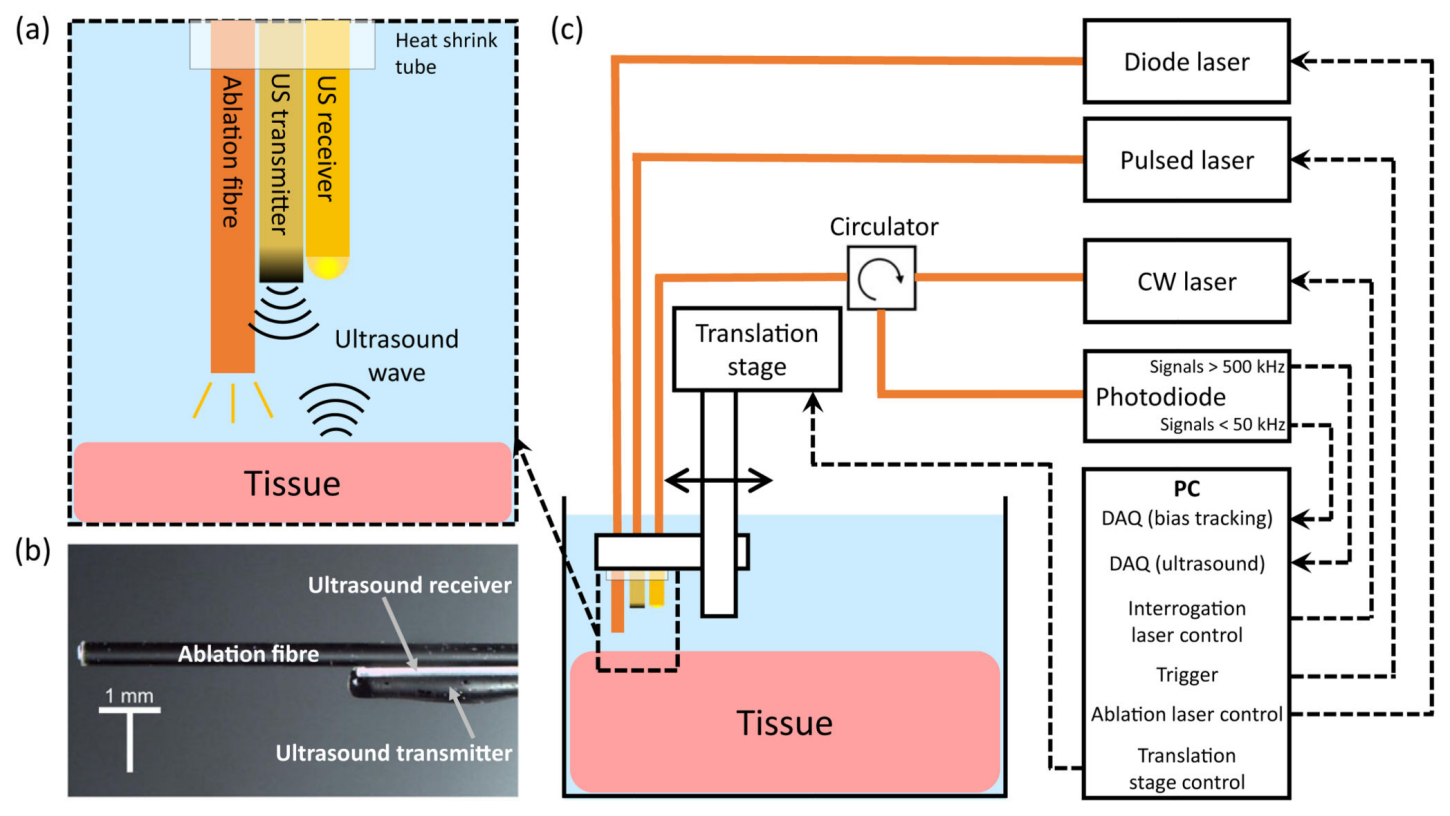
Schematic overview of the dual-modality OpUS imaging and laser ablation system. **a** The schematic of the fibre device (ultrasound generation, reception and ablation fibres). **b** The corresponding microscope image of the fibre device. **c** OpUS Imaging and ablation system setup including the fibre device and console. CW laser, continuous wave laser; DAQ, data acquisition.

The all-optical ablation and ultrasound imaging system comprised two parts; an optical fibre device for ultra-sound imaging and delivery of ablation light (Fig. 5a, b), and a console to control and interrogate the device (Fig. 5c). The all-optical fibre device comprised three optical fibres; one to transmit ultrasound, one to receive ultrasound, and one to deliver light for laser ablation.

The optical ultrasound transmitter had a 400 *μ*m aperture and used a composite material consisting of a near-infrared absorbing dye (Epolight 9837, Epolin, USA) and PDMS (MED-1000, Polymer Systems Technology, UK) for ultrasound generation. The detailed process for producing the composites and applying it to the fibre end face were described previously^38^. Briefly, the near-infrared absorbing dye (10 mg) was mixed with xylene (0.5 ml), and subsequently PDMS (0.25 g) was incorporated and manually stirred until homogeneous. The prepared optical fibre was manually dipped into the dye-PDMS solution to form a coating layer on the distal end face. The coating was left in ambient conditions for 24 h to cure. The fabricated transmitter generated ultrasound pressures in excess of 1.5 MPa, with corresponding bandwidth of more than 20 MHz, allowing centimetre depth signal penetration on tissue and high imaging resolution^38^. Previous studies have shown that these ultrasound generating coatings are stable for continuous use over extended periods (ca. 1 h)^58^, much longer than those used during these studies.

The optical ultrasound receiver comprised a plano-concave microresonator located on the distal tip of a single mode optical fibre. The cavity design and fabrication have been reported in previous work^46,48^. In brief, one dielectric mirror with a reflectivity of 98% was deposited directly onto the fibre, then the fibre was dip coated with an epoxy dome, followed by coating with a second dielectric mirror. The dielectric mirrors were sputtered coated following the method previously described by Zhang et al.^64^ Subsequently, a Parylene C overcoat was added to the fibre distal end for protection. Ultrasound waves reflected from tissue impinge on the plano-concave microresonator, changing the cavity thickness and therefore its reflectivity, which was monitored using the console. These sensors have been shown to provide high bandwidths and sensitivities (noise equivalent pressure < 100 Pa over a bandwidth of 20 MHz)^46,48^.

The optical fibre for laser ablation comprised a polyimide clad fused silica core/ cladding optical fibre with a 400 *μ*m core diameter (WF 400/440/470 P, CeramOptec, Germany). The distal end was cleaved perpendicular to the optical fibre axis to provide a uniform surface for light emission. Emitted laser light was directed ahead of the optical fibre with the divergence defined by the numerical aperture of the optical fibre (0.22 NA).

The ultrasound console used to control and interrogate the device comprised several components (Fig. 5c). A Q-switched Nd:YAG laser (SPOT-10-500-1064, Elforlight, UK) with wavelength 1064 nm, a pulse width 2 ns, and a repetition rate of 100 Hz was used for ultrasound excitation. The pulse energy provided to the coating for ultrasound generation was 30.1 *μ*J, corresponding to a fluence of 24.0 mJ/cm^2^.

For ultrasound reception, a continuous wave tuneable laser (Tunics T100S-HP CL, Yenista Optics, France), operating in the wavelength range 1500–1600 nm, was used to interrogate the plano-concave microresonator. The laser was connected via a fibre optic circulator, which allowed the reflected signal to be detected with a custom photoreceiver. The photoreceiver split the signal into low-frequency (<50 kHz) and high-frequency (>500 kHz) components. The low-frequency part was digitised at 16 bits (PCI-6251, National Instruments, UK) and was used to record the plano-concave microresonator transfer function and to estimate the optimum bias point. This signal varies slowly with time and thus only requires the low-frequency component of the received optical signal. The high-frequency part was used to record the modulation of reflected optical power induced by the incident acoustic wave and was digitised at 14 bits with a sample rate of 100 MS/s (PCI-5142, National Instruments, UK). The received ultrasound signal is typically high frequency, thus by high pass filtering at 500 kHz noise from lower frequencies is removed. The sensor was biased to the point of the maximum derivative of the interferometer transfer function (ITF) of the plano-concave microresonator to optimise the sensitivity^64^. In this case, the acoustically induced cavity thickness can be converted to a corresponding modulation of the reflected optical power, by which the reflected ultrasound wave incident on the fibre distal tip can be derived.

For laser ablation, a diode laser (Axcel Photonics Inc, US) providing 808 nm wavelength light was employed to irradiate tissue via the ablation fibre. The laser had a maximum power output of 6 W and was controlled via a driver (LDP-CW 18-05, PicoLAS, Germany) with a custom LabVIEW (National Instruments, USA) programme, providing variable laser output power and duration. The power delivered by the fibre was measured and calibrated by an external power metre.

Within the all-optical fibre device, the ultrasound transmitter, receiver and ablation fibre were held together by heat shrink tube. The distal end face of the ultrasound transmitter and receiver were aligned on the same level, while the ablation fibre was advanced 5 mm from them to avoid heating of the plano-concave microresonator during the ablation procedure. The whole probe was mounted on a motorised stage (MTS50/M-Z8, Thorlabs, UK) and its distal end was submerged in a water bath, containing saline, for imaging (Fig. 5).

### Optical ablation protocol

The optical ablation operation and monitoring were performed on ex vivo porcine heart and liver tissue. The fresh porcine heart and liver were obtained from a local butcher from an animal slaughtered on the day of the experiment and were stored at 5 °C prior to the experiment. During the experiment, the tissue was sectioned (40 × 20 × 10 mm) and affixed on to a cork ring in the water bath.

In this work, two ablation regimes were used; contact and non-contact. In the non-contact regime a gap was maintained between the optical ablation fibre and the tissue surface during the procedure. While for the contact regime there was direct contact between the optical ablation fibre and the tissue surface during the procedure.

Regarding the non-contact regime, the dual-modality device was mounted above the tissue with a 1 mm gap between the tissue surface and ablation fibre distal tip such that carbonised or ejected tissue formed during tissue ablation did not adhere to the fibre tip. Ablation powers of 3.5, 4.0 and 4.5 W, with exposure times of 30, 45 and 60 s for each power, were used. These parameters, which ensured that tissue only underwent the coagulative necrosis phase, were determined empirically with pre-experiments on ex vivo porcine heart and liver tissue. For each laser power-duration combination, three repetitions were performed using different positions on the same section of tissue. This was realised using a motorised stage (MTS50/M-Z8, Thorlabs, UK) to control the device location. The power and duration setup were lower than previously reported for in vivo laser ablation^65^ since the absence of heat convection due to blood perfusion can result in higher temperature over the tissue^66^.

Regarding the contact regime, the ablation fibre was contacted with the tissue surface. A single ablation power of 1 W was delivered with 45 s duration. This laser power and duration was chosen empirically to maintain the function of the plano-concave microresonator and avoid rapid carbonisation of tissue.

On the completion of ablation procedures, the tissue was sliced through the centre of the ablation lesions to enable the lesion depth visualisation and measurement by stereomicroscopy (Leica, UK). Stereomicroscope ablation depths were determined by taking the average measurements of three human observers. The lesion depths were measured independently without knowledge of the OpUS depth measurements, based on the visual inspection of thermal-induced lesion colour changes compared to the surrounding tissue.

### M-mode lesion imaging

All-optical M-mode ultrasound imaging was performed during ablation to track lesion depth. A custom programme based on LabVIEW (National Instruments, USA) was used for data acquisition, providing data processing and real-time display of the M-mode ultrasound images. For M-mode imaging, A-lines were acquired at a rate of 100 Hz and acquired data was saved for off-line processing. The acquired data was processed in several steps prior to display and segmentation. Firstly, the acquired A-lines were bandpass filtered (Butterworth, ^10–40^ MHz, 4th order) for noise suppression. Subsequently, a Hilbert transform was applied to obtain the signal envelope, followed by a log transform for display. Individual A-lines were concatenated in time to produce the M-mode image.

**Fig. 6:**
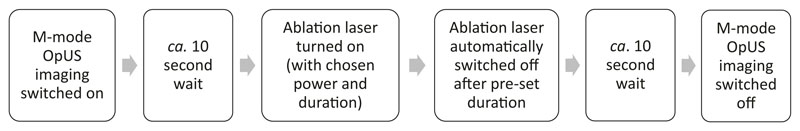
Flow diagram of the operational workflow. The operational workflow for ablation lesion imaging is outlined, including initiation of M-mode OpUS imaging, followed by laser ablation and monitoring.

M-mode image acquisition was started 10 s prior to turning the ablation laser on, this provided a clear view about the signal change during laser ablation. Further, image acquisition was continued for 10 s after completion of the laser ablation, allowing measurement of the resulting lesion depth (Fig. 6).

### Lesion depth tracking

The lesion depth was measured on the acquired M-mode images. Tissue exposed to laser ablation exhibited increased brightness on the M-mode ultrasound image compared to normal tissue. Therefore, the automated lesion depth tracking was developed on the basis of image segmentation. Prior to segmentation, the noise on ultrasound images was suppressed by a moving average kernel. The algorithm used for segmentation was fuzzy c-means (FCM), integrating local intensity and spatial information to increase robustness to noise and artefacts^67^.

Briefly, the FCM algorithm mapped each pixel into multiple clusters by introducing a membership function that represented the probability of each pixel belonging to a specific cluster. The cost function used in FCM was subjected as follows^67,68^:

(1) $$J=\sum_{n=1}^{N}\sum_{m=1}^{C}\mu_{mn}∥i_{n}-v_{m}{∥}^{2}$$

where *N* was the number of pixels; *C* was the number of clusters; *i_n_* was the specific pixel; *v_m_* was the centroid of *m*^th^ cluster and ∥·∥ denoted the norm operation. In order to minimise the cost function, the membership functions *μ*_*mn*_ and *v_m_* were updated iteratively as follows^68^:

(2) $$\mu_{mn}=\frac{∥i_{n}-v_{m}{∥}^{-2/(p-1)}}{\sum_{k=1}^{C}∥i_{n}-v_{k}{∥}^{-2/(p-1)}}$$

(3) $$v_{m}=\frac{\sum_{n=1}^{N}\mu_{mn}^{p}i_{n}}{\sum_{n=1}^{N}\mu_{mn}^{p}}$$

where *p* was the parameter controlling the fuzziness of the segmentation. By incorporating the spatial information *h_mn_*, the membership functions were updated to:

(4) $${\mu^{\prime }}_{mn}=\frac{\mu_{mn}h_{mn}}{\sum_{k=1}^{c}\mu_{kn}h_{kn}}$$

the *h_mn_* presented spatial information by:

(5) $$h_{mn}=\sum_{k\in N_{n}}\mu_{mk}$$

where *N_n_* denoted the spatial window centred around the pixel *n*. The iteration stopped when the maximum difference between the cluster centres derived from two consecutive iterations was less than a threshold value (0.02). After the convergence of the cost function, each pixel was assigned to the specific cluster where the membership was maximal. Following the segmentation, the morphological operators including image opening and closing were applied for extracting the ablation region.

### Data analysis

To assess whether the OpUS imaging could delineate the ablation depth accurately, the OpUS measurements were compared with ablated depth measurements acquired by stereomicroscopy. In terms of the non-contact regime, the Wilcoxon signed-rank test (two-tailed, *p* < 0.05) were performed between the depth measurements of OpUS and stereomicroscopy for all the resulting lesions caused by each laser power-duration combination and their repeats. The Wilcoxon signed-rank test was not employed on the contact regime since the lower number of samples would lead to invalid statistical results and the purpose of these experiments was to highlight the visualisation of carbonisation in the M-mode imaging.

## Data Availability

Data underlying the results presented in this paper are not publicly available at this time but may be obtained from the authors upon reasonable request.

## Code availability

MATLAB Code used in this study to track the lesion depth is available at the online repository Zenodo at https://doi.org/10.5281/zenodo.6684276.
